# METTL3-mediated m6A modification of SLC7A11 enhances nasopharyngeal carcinoma radioresistance by inhibiting ferroptosis

**DOI:** 10.7150/ijbs.100518

**Published:** 2025-02-10

**Authors:** Zili Dai, Baisheng Lin, Maohua Qin, Yunen Lin, Li Wang, Kai Liao, Guofeng Xie, Feixiang Wang, Jian Zhang

**Affiliations:** 1 Department of Radiation Oncology, Guangzhou Institute of Cancer Research, the Affiliated Cancer Hospital, Guangzhou Medical University, Guangzhou 510095, China.; 2 Department of pathology, Guangzhou Institute of Cancer Research, the Affiliated Cancer Hospital, Guangzhou Medical University, Guangzhou, China.; 3 Department of Thoracic Surgery, Guangzhou Institute of Cancer Research, the Affiliated Cancer Hospital, Guangzhou Medical University, Guangzhou, China.

**Keywords:** nasopharyngeal carcinoma, m^6^A modification, SLC7A11, radiosensitivity, ferroptosis

## Abstract

Radiotherapy is the primary treatment for nasopharyngeal carcinoma (NPC); nonetheless, radioresistance remains the leading cause of localized recurrence. Our study demonstrates a significant increase in the N6-methyladenosine (m6A) methylase METTL3 in NPC and other tumors. Mechanistically, METTL3 acts as an m6A methylase, enhancing the m6A modification of the solute carrier family 7 member 11 (SLC7A11) transcript, which increases its stability and expression, thereby inhibiting radiation-induced ferroptosis and ultimately inducing radioresistance in NPC. Furthermore, silencing SLC7A11 or employing the ferroptosis inducer Erastin negated the promoting effect of METTL3 on NPC cell radioresistance. These findings suggest that METTL3 could be a novel therapeutic target for overcoming radiotherapy resistance in NPC.

## 1. Introduction

Nasopharyngeal carcinoma (NPC), a malignancy originating from the epithelium of the nasopharyngeal mucosa, can be highly locally invasive and prone to distant metastases. NPC is highly prevalent among people from Southeast Asian regions, especially Southern China 1, 2. With the application of advanced radiotherapeutic techniques and concurrent chemoradiotherapy, the 5-year overall survival rate for NPC patients has surged from 17-35% to over 75% 3. Nevertheless, approximately 10-20% of cases exhibit intrinsic resistance to radiotherapy, leading to local recurrence and distant metastasis 4, 5. Therefore, it is significant to clarify the molecular mechanisms underlying NPC radioresistance to develop novel therapeutic strategies.

Epigenetic alterations have been identified as key genetic features in tumorigenesis and progression, and their reversible nature makes them potential targets for overcoming treatment resistance 6, 7. N6-methyladenosine (m6A) is the most prevalent dynamic modification of RNA methylation, involved in crucial cellular processes such as RNA metabolism, splicing, translation, nuclear export, and transport 8-11. We previously employed pyrophosphate sequencing to confirm that 70% of genes in NPC are hypermethylated 12. METTL3 is significantly overexpressed in head and neck tumors and is linked to poor patient prognosis. Through screening m6A-related proteins, we discovered that the m6A methyltransferase METTL3 mediated the reduced expression of ZNF750, which activated the FGF14 signaling pathway and promoted the proliferation and invasion of NPC cells 13. Simultaneously, we confirmed that METTL3 stabilized the non-coding lncRNA FAM225A, which functions as a ceRNA to upregulate ITGB3 and promotes the proliferation of NPC. This process promotes the proliferation, migration, and invasion of NPC 14. Recently, several studies have clarified that N6-methyladenosine (m6A) methylase (METTL3) plays oncogenic roles in radiosensitivity in glioblastoma 15, gastric cancer 16, and hypopharyngeal squamous cell carcinoma 17, representing an opportunity for the development of METTL3-targeted therapies for NPC.

Ferroptosis, a new type of programmed cell death, is characterized by iron accumulation and lipid peroxidation during the cell death process 18. Recent studies have identified that ferroptosis is closely related to the pathophysiological processes of many diseases, including cancers 18-20. Targeting ferroptosis-associated proteins for direct induction or secondary regulation has emerged as an effective therapeutic approach to triggering tumor cell demise, particularly in malignant tumors resistant to radiation 21, 22. However, the underlying role of ferroptosis in NPC radiosensitivity remains unclear.

In this study, we found an upregulation of METTL3 in various cancers, including NPC, which is associated with poor survival. Further *in vitro* and *in vivo* investigations indicate that METTL3-mediated m6A modification of SLC7A11, dependent on IGF2BP2, maintains its stability, inhibits NPC ferroptosis, and promotes radioresistance. Hence, METTL3 may serve as a predictive marker and therapeutic target for radioresistance in NPC.

## 2. Materials and methods

### 2.1 Clinical specimen collection

NPC specimens (n=78) were collected from the Affiliated Cancer Hospital of Guangzhou Medical University (Guangzhou, China) and prepared for immunohistochemical staining experiments. Fresh frozen biopsy specimens of NPC (n=8) and nasopharyngeal epithelial specimens (n=8) were collected for protein extraction. None of the patients had received any anti-tumor therapy prior to specimen collection. This study was approved by the Ethics Review Committee of the Affiliated Cancer Hospital of Guangzhou Medical University and adhered to the guidelines of the Helsinki Declaration.

### 2.2 Cell culture

The normal human nasopharyngeal epithelial cell line NP69 was cultured in KSFM keratinocyte growth medium (Elgbio) supplemented with KSFGS cell growth factor. HEK-293T cells provided by the American Type Culture Collection (ATCC) were cultured in DMEM medium (Gibco) supplemented with 10% fetal bovine serum (FBS). Human NPC cell lines (HONE1, SUNE1) were cultured in RPMI-1640 medium (Gibco) supplemented with 10% FBS. All normal nasopharyngeal epithelial cell lines and NPC cell lines were generously provided by Professor Musheng Zeng (Sun Yat-sen University Cancer Center). HEK-293T cells were obtained from ATCC and cultured in DMEM medium (Invitrogen). Additionally, all cells were cultured at 37°C with 5% CO2. Negative results were obtained for all cells in mycoplasma testing, and cell lines were not passaged for more than three months.

### 2.3 Transient transfection and establishment of stable cell lines

The plasmids pEnter-IGF2BP1-Flag, pEnter-IGF2BP2-Flag, pEnter-IGF2BP3-Flag, pEnter-YTHDF1-Flag, pEnter-YTHDF2-Flag, pEnter-YTHDF3-Flag, pEnter-SLC7A11-Flag, and the corresponding pEnter-vector plasmids were purchased from WZ Biosciences. Plasmid DNA was purified using a plasmid extraction kit (TIANGEN). Transient transfection experiments were performed using the Lipofectamine 3000 reagent kit (Invitrogen) according to the manufacturer's instructions.

The plasmids Plko-u6-METTL3-gfp-puro, Plko-u6-METTL3-gfp-puro-vector, pCDH-METTL3-puro, and pCDH-METTL3-puro-vector were purchased from Getein Biotech. The catalytic mutant METTL3 (aa395-398, from DPPW to APPA) was generated by site-directed mutagenesis. To produce cells capable of stably expressing METTL3 or the vector, HEK-293T cells were co-transfected with lentiviral packaging plasmids and either pCDH-METTL3-puro or pCDH-METTL3-puro-vector. The collected lentiviruses were then used to infect NPC cells. Stable cells were selected using 2 μg/ml puromycin (Solarbio) and verified by qRT-PCR and Western blotting.

### 2.4 Western blot assay

Total cellular proteins were extracted using RIPA lysis buffer (CWBIO) supplemented with phosphatase and protease inhibitors (Beyotime). The protein concentration was measured using a protein quantification kit (CWBIO), and then the proteins were denatured. Equal amounts of protein (30 μg/sample) were separated by 5-15% sodium dodecyl sulfate-polyacrylamide gel electrophoresis (SDS-PAGE) and transferred to a PVDF membrane (Millipore). The membrane was blocked with 5% bovine serum albumin (BSA) in TBST (1% Tween-20 in Tris-buffered saline) at room temperature for 1 hour. The membrane was then incubated with the primary antibodies against the target proteins overnight at 4°C (Table S2). The membrane was incubated with the corresponding HRP-conjugated secondary antibodies for 1-2 hours at room temperature. The immunocomplexes were detected using a chemiluminescence reagent (EpiZyme), with GAPDH as the loading control.

### 2.5 RNA extraction and quantitative Real-Time PCR (qRT-PCR)

Total RNA was extracted using RNAiso Plus (Takara) according to the manufacturer's instructions. The concentration of total RNA was measured using a NanoDrop spectrophotometer. Total RNA was reverse-transcribed into cDNA using Evo M-MLV reverse transcriptase pre-mixture (Accurate Biology). qRT-PCR samples were prepared using a SYBR Green reagent kit (Accurate Biology) and then quantitatively detected using a real-time fluorescence quantitative PCR instrument (Roche). Human GAPDH was used as an internal control for normalization. Primer sequences are provided in Table S1.

### 2.6 Cell viability assay

The CCK-8 cell counting kit (GLPBIO) was used to evaluate cell viability. NPC cells (5 × 10^3^ cells/well) were seeded in 96-well plates and exposed to 2 Gy, 4 Gy, 6 Gy, and 8 Gy of radiation. After 24 hours of incubation, 100 μl of RPMI-1640 medium containing 10% CCK-8 solution was added to each well, and the cells were further incubated for 2 hours. The absorbance was then measured at 450 nm using a spectrophotometer (Tecan).

### 2.7 Colony formation assay

Cells in the logarithmic growth phase were trypsinized and resuspended into a single-cell suspension. Cells (400-1000 cells/well) were seeded in 6-well plates. After 24 hours, the cells were exposed to different doses of X-ray radiation as required by the experiment. The cells were then cultured for 14 days, washed once with PBS, fixed with 4% paraformaldehyde, and stained with crystal violet.

### 2.8 RNA stability assay

Cells were seeded in 12-well plates and treated with actinomycin D (5 μg/mL, CST) for 0, 0.5, 1, 1.5, and 2 hours. RNA was extracted at the specified time points and analyzed by quantitative PCR. The half-life of the mRNA was calculated using linear regression analysis.

### 2.9 Glutathione (GSH) Quantification and Malondialdehyde (MDA) Determination

The total cellular glutathione level was detected using GSH and GSSG Assay Kit (Beyotime) in accordance with the manufacturer's instructions, and the absorbance at 412 nm was measured using a spectrophotometer. For MDA detection, the cells were lysed and treated with Lipid Peroxidation MDA Assay Kit (Beyotime). The absorbance at 532 nm was measured.

### 2.10 Quantification of intracellular ferrous ions (Fe^2+^)

According to the instructions, the total cellular Fe2+ level was detected using Cell Ferrous Iron Colorimetric Assay Kit (Elabscience). The collected cells were lysed by adding 0.2 mL of Reagent I per 1×10^6^ cells, mixed well, and incubated on ice for 10 minutes. After centrifugation at 15,000×g for 10 minutes, the supernatant was used for subsequent measurements. Reagent II was added to the standard wells, test wells, and control wells, mixed well, and incubated at 37°C for 10 minutes. The absorbance was measured at 593 nm using a spectrophotometer.

### 2.11 Quantification of Reactive Oxygen Species (ROS)

According to the instructions, ROS Assay Kit (Beyotime) was used to detect intracellular ROS levels. DCFH-DA was diluted 1:1000 in serum-free culture medium to achieve a final concentration of 10 μM. Cells were seeded at a density of 2 × 10^5^ cells per well in a 6-cm dish and cultured for 12 hours. The cell culture medium was then replaced with either drug-free medium (control group), drug-containing medium, or medium treated with 4 Gy X-ray irradiation, and incubated for an additional 24 hours. The cells were trypsinized, collected, and resuspended in the diluted DCFH-DA solution at a concentration of 10^6^-10^7^ cells/mL, then incubated at 37°C in a cell culture incubator for 20 minutes.

### 2.12 Immunohistochemistry (IHC) staining and hematoxylin and eosin (H&E) Staining

Paraffin-embedded NPC tissues were sectioned at a thickness of 4 μm. After incubation at 65°C for 2 hours, the tissue sections were deparaffinized in xylene and rehydrated through a graded ethanol series. Endogenous peroxidase activity was blocked using 4% hydrogen peroxide. Normal goat serum was used to block the tissue. The tissue was incubated with the primary antibody overnight at 4°C (Table S2). The tissue was incubated with the secondary antibody for 20 minutes and stained with 3,3'-diaminobenzidine (DAB) substrate (ZSGB-BIO). The percentage of positive tumor cells and their staining intensity were evaluated. According to a semi-quantitative system, the staining intensity was scored as 0 (no staining), 1 (weak), 2 (moderate), or 3 (strong). The percentage of positive tumor cells was scored as 1 (<25%), 2 (26-50%), 3 (51-75%), or 4 (>75%). The percentage of positive cells was determined from five different random areas. The staining intensity score was multiplied by the positive cell percentage score to quantify the expression and obtain the final score. For H&E staining, the sections were immersed in hematoxylin for 5 minutes, washed in water for 5 minutes, and then stained with eosin for 5-10 minutes. After dehydration in different concentrations of ethanol, the sections were mounted with coverslips. The stained tissue sections were imaged using a slide scanner microscope (3DHISTECH).

### 2.13 Immunofluorescence (IF) staining

Cell suspensions (1-2 × 10^3^ cells/mL) were seeded onto cell slides, fixed with 4% paraformaldehyde for 15 minutes, permeabilized with 0.5% Triton X-100 for 10 minutes, and blocked with 5% BSA for 30 minutes. The cells were then incubated with the primary antibodies overnight at 4°C (Table S2). The following day, the cells were incubated with the corresponding fluorescent-labeled secondary antibodies for 1 hour at room temperature. The cells were then counterstained with an anti-fluorescence quenching mounting medium containing DAPI for nuclear staining. The slides were observed using a laser scanning confocal microscope (ZEISS).

### 2.14 Transmission electron microscopy (TEM)

The cells were subjected to routine trypsin digestion, which was terminated using complete culture medium. The cell suspension was then centrifuged at 1,000 rpm for 5-10 minutes to collect the cell pellet. The supernatant was discarded, and the compact cell pellet was gently resuspended in 1 mL of 2.5% glutaraldehyde fixative at room temperature. The cells were fixed in the dark for 15 minutes at room temperature. After post-fixation with 1% osmium tetroxide and dehydration, the samples were embedded in EPON resin. The embedded samples were sectioned and stained with uranyl acetate. The stained sections were then examined under a transmission electron microscope.

### 2.15 M6A immunoprecipitation sequencing (MeRIP-seq) and mRNA sequencing (mRNA-seq)

MeRIP-seq and mRNA-seq experiments were conducted by Gene Denovo Biology, utilizing the Illumina NovaSeq 6000 sequencing system. The same company also assisted with subsequent bioinformatics analyses. The m6A peaks were visualized using the UCSC genome browser or IGV software. Motif discovery, both de novo and known, was performed with HOMER, followed by motif localization using Perl scripts. FPKM (fragments per kilobase of transcript per million) values for all mRNAs in the input libraries were calculated using StringTie. Gene Set Enrichment Analysis (GSEA) was conducted using the R package clusterProfiler.

### 2.16 RNA immunoprecipitation (RIP)

RIP detection was conducted using the RIP reagent kit according to the manufacturer's protocol (Boxin Biotech, China). Cells were lysed to remove DNA, and the resulting lysate was divided into input, IgG, and IP groups. In the IgG and IP groups, 5 μg of IgG and primary antibody were added (Table S2), and the samples were incubated overnight at 4°C. The following day, 20 μL of Protein A/G magnetic beads were added to the IgG and IP groups, and the samples were incubated for 1 hour at 4°C. RNA-protein complexes bound to the magnetic beads were then eluted. RNA was extracted and purified using a phenol-chloroform-isoamyl alcohol solution. RT-qPCR was performed to detect the expression levels of the target gene.

### 2.17 Animal experiments

Five-week-old female BALB/c nude mice were purchased from the Guangdong Medical Laboratory Animal Center. HONE1 cells (1×10^7^ cells) were mixed with Matrigel (1:1, Corning) and subcutaneously injected into the fat pad of the nude mice. After palpable tumors formed, the mice were randomly divided into six groups: Ctrl group, IR group (4 Gy), and IR (4 Gy) combined with the ferroptosis inducer Erastin (10 mg/kg; ip) group. Tumor volumes were measured every three days and calculated as volume = length × width^2^/2. The primary tumor cells were stained with primary antibody (Table S2).

### 2.18 Database analysis

The METTL3 transcriptome data were downloaded from the TCGA database (https://tcga-data.nci.nih.gov/tcga/) and the Gene Expression Omnibus (GEO) database (https://www.ncbi.nlm.nih.gov/geo/). Patients with incomplete clinicopathological information were excluded from data curation and analysis. The gene expression data from the microarray were normalized using Transcripts Per Million (TPM).

### 2.19 Statistical analysis

All experiments were performed at least three times. Data were statistically analyzed using Prism 8.0 software. An unpaired t-test was used to analyze the differences between two groups. One-way ANOVA was used for comparisons among multiple groups. Kaplan-Meier survival curves were plotted, and the log-rank test was used for comparison. The results are presented as mean ± standard error of the mean (mean ± SEM).* p < 0.05* was considered statistically significant.

## 3. Results

### 3.1 METTL3 is upregulated and correlates with poor prognosis in NPC

**Figure 1:**
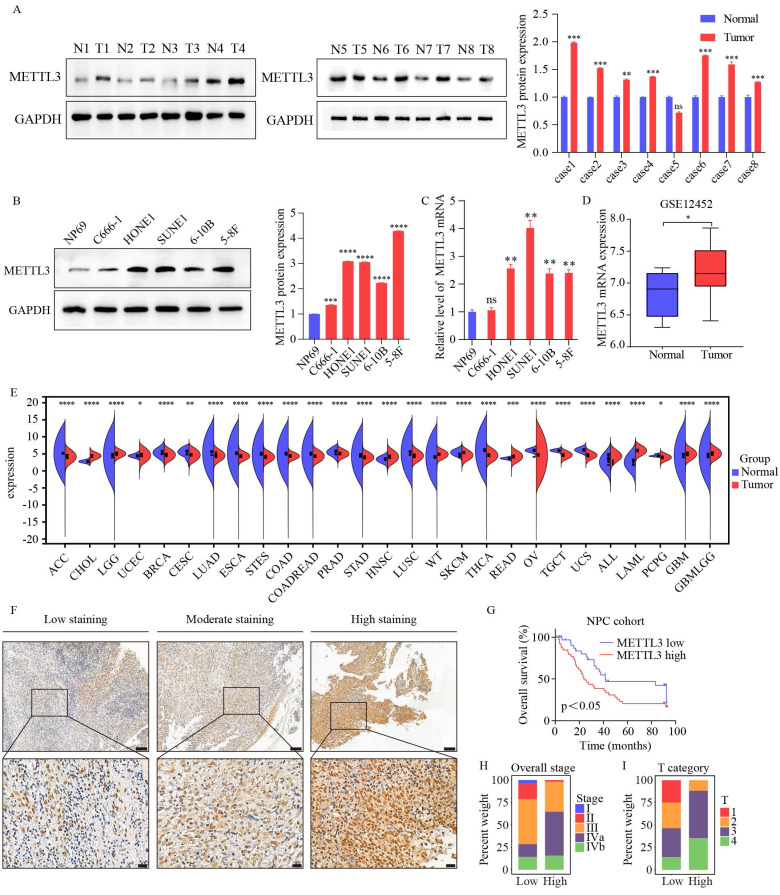
** Elevated expression of METTL3 associated with poor prognosis in NPC patients.** (A) Differential expression of METTL3 in normal nasopharyngeal epithelial tissues and NPC tissues (N: normal; T: tumor). The right panel displays quantification data from three replicates of WB assay. (B) Protein expression of METTL3 in normal and NPC cell lines. The right panel shows quantification data from three replicates of Western blot analysis. (C) Expression of METTL3 mRNA in nasopharyngeal normal cell line (NP69) and NPC cell lines. (D) Differential expression of METTL3 mRNA in the GSE12452 dataset (N=10, T=31). (E) Expression of METTL3 in various cancer types in TCGA data. (F) Representative IHC staining images of METTL3 expression in NPC tissues. (G) Kaplan-Meier analysis of overall survival rates of NPC patients with high/low expression of METTL3. (H-I) Distribution of patients with high and low expression of METTL3 in tumor grade (H) and comparison of T stage (I). Data are presented as mean ± SEM of three independent experiments, * *p* < 0.05; ** p < 0.01; *** p < 0.001.

To elucidate the role of METTL3 in NPC, we first assessed METTL3 expression in eight paired clinical samples. The results revealed a marked upregulation of METTL3 in NPC tissues (Figure 1A). However, we found no significant difference in METTL3 expression in the fifth tissue pair. This finding suggests potential tissue-specific variation and aligns with the broader biological context we are investigating. Compared to the nasopharyngeal epithelial cell line NP69, METTL3 was also upregulated in NPC cell lines (Figure 1B-C). Meanwhile, GEO (GSE12452) showed that the mRNA levels of METTL3 were markedly upregulated in NPC (Figure 1D). TCGA database reveals differential expression of METTL3 mRNA across 27 solid tumor types, with notably higher expression in HNSC (Figure 1E). Survival analysis indicated that METTL3 was associated with poor survival (Figure 1F and G). Subgroup analysis indicated that higher METTL3 expression was significantly associated with clinical stage and T stage (p<0.05, Figure 1H-I, Table S3). Collectively, our data indicated that METTL3 may act as an oncogenic factor in the progression of NPC.

### 3.2 METTL3 suppresses NPC radiosensitivity *in vitro* and *in vivo*

**Figure 2:**
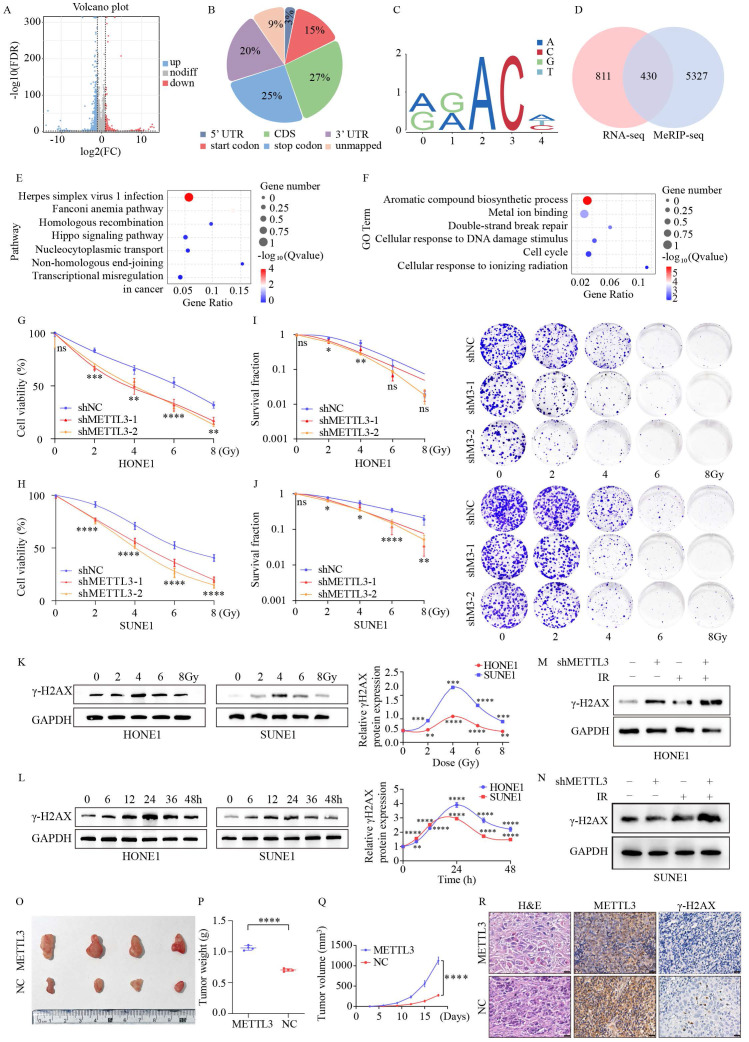
** METTL3 facilitates radiotherapy resistance both *in vitro* and *in vivo*.** (A) Volcano plot illustrates the RNA-seq results of differentially expressed genes in control and METTL3-overexpressing HONE1 cells. Blue dots represent significantly upregulated genes, red dots indicate significantly downregulated genes, and gray dots denote genes without significant differential expression (|log2FC| ≥ 2, P-value ≤ 0.05). (B) Pie chart shows the distribution of m6A peaks in different gene regions in METTL3-overexpressing HONE1 cells, including CDS (27%), stop codon (25%), 3' UTR (20%), start codon (15%), unmapped (9%), and stop codon (3%). (C) The sequence logo displays the enriched consensus sequence around the m6A peaks. (D) Venn diagram shows the overlap between differentially expressed genes identified by RNA-seq and m6A-modified genes identified by MeRIP-seq. (E-F) KEGG pathway enrichment analysis (E) and Gene Ontology (GO) term enrichment analysis (F) of overlapping differentially expressed genes (DEGs). (G-J) Cell viability (G-H) and clonogenic capability (I-J) of NPC cells with METTL3 knockdown after exposure to different doses of irradiation (0, 2, 4, 6, 8 Gy). (K-L) Temporal dynamics of γ-H2AX levels in NPC cells following treatment with varying doses of irradiation, as assessed by WB. (M-N) Alterations in γ-H2AX levels after irradiation (4 Gy) for 24 hours in control and METTL3 knockdown cells. (O) Enhanced growth of subcutaneous xenografts in nude mice upon METTL3 overexpression (n=4). (P) Tumors weight. (Q) The growth curve of tumors. (R) Histological examination of tumor sections stained with H&E, along with IHC staining using anti-METTL3 and anti-γ-H2AX antibodies (scale bar = 20 µm). Data represent the mean ± SEM of three independent experiments, * *p* < 0.05; ** p < 0.01; *** p < 0.001.

To identify potential targets regulated by METTL3, we conducted RNA sequencing (RNA-seq) on HONE1 cells with overexpressed METTL3. Overexpression of METTL3 caused dysregulated gene expression, resulting in 943 genes being upregulated and 2201 genes being downregulated (Figure 2A). Subsequently, using m6A-seq technology, we mapped the m6A methylome in NPC cells overexpressing METTL3, identifying 25,070 m6A peaks across 20,332 genes. These m6A modifications were mainly enriched in protein-coding transcripts (27%), stop codons (25%), and 3'-UTRs (20%) (Figure 2B). In line with previous studies, GGAC [U/A] was identified as the consensus sequence for m6A in HONE1 cells (Figure 2C). By integrating m6A-seq and RNA-seq data, we identified 430 overlapping differentially expressed genes (DEGs) with m6A modifications (Figure 2D). KEGG and GO analyses revealed that the overlapping DEGs were significantly enriched in pathways and processes related to radiation damage and ferroptosis, such as DNA damage repair, homologous and non-homologous recombination, biosynthesis of aromatic compounds, and metal ion binding (Figure 2E-F).

To investigate the impact of METTL3 on NPC, we downregulated METTL3 expression in NPC cells using shRNA and validated it through Western blot (WB) and quantitative PCR (qPCR) assays (Figure S1A-B). CCK-8 assays revealed that METTL3-KD significantly decreased the survival ability of NPC cells after IR treatment (Figure 2G-H). METTL3 knockdown reduced the proliferative capacity of the cells (Figure S1C). METTL3-KD decreased the clonogenic capacity of NPC cells (Figure 2I-J). To determine the optimal time and dosage for radiation, NPC cells were treated with different doses and durations. The results demonstrated that γ-H2AX expression was dose-dependent and time-dependent, particularly at 4 Gy for 24 hours (Figure 2K-L). METTL3-KD contributed to an upregulation of γ-H2AX expression, which was significantly enhanced when combined with IR treatment (Figure 2M-N). Likewise, IF staining revealed a pronounced increase in the number of γ-H2AX foci in METTL3-KD cells 24 hours post-exposure to 4 Gy irradiation (Figure S1D-E). Xenograft tumor models indicated that METTL3 promoted NPC growth *in vivo* (Figure 2O-Q). IHC analysis revealed that METTL3 could decrease γ-H2AX expression (Figure 2R). These findings suggest that METTL3 is associated with NPC malignant features, such as radioresistance.

### 3.3 METTL3 inhibits NPC radiation-induced ferroptosis

Radiotherapy uses high-energy ionizing radiation (IR) to produce DNA double-strand breaks, inducing cell cycle arrest, senescence, and various forms of cell death, including apoptosis, necrosis, and autophagy. In addition to directly damaging DNA, IR induces indirect cellular effects by generating reactive oxygen species (ROS) such as hydroxyl radicals and hydrogen peroxide, which further contribute to DNA damage 23.

**Figure 3:**
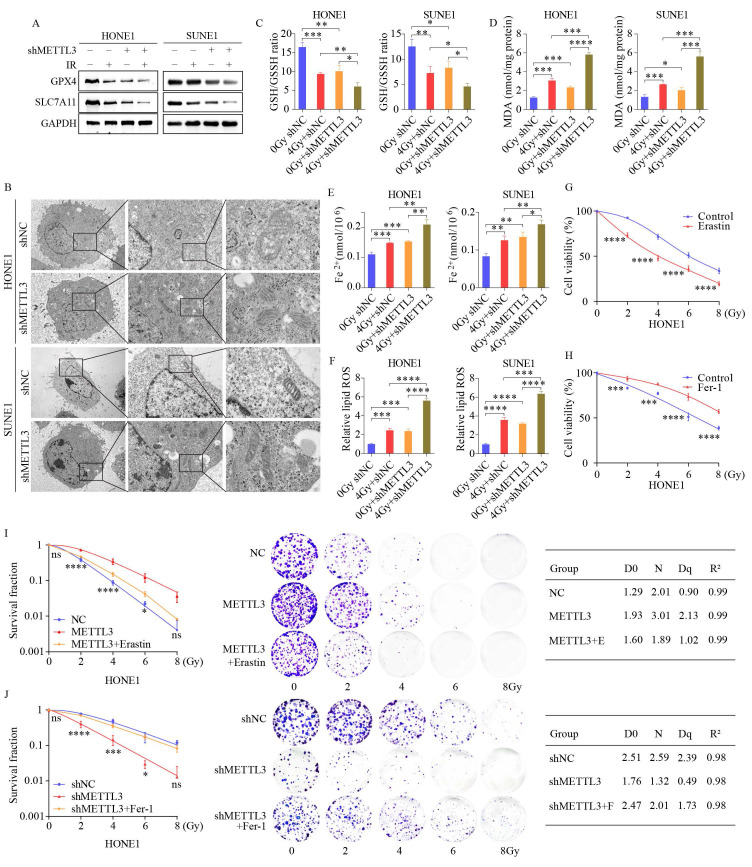
** METTL3 knockdown sensitizes NPC cells to radiotherapy by promoting ferroptosis.** (A) WB images depicting key ferroptosis-associated proteins in cells with or without irradiation (IR) treatment upon METTL3 knockdown. (B) TEM revealing distinctive morphological features of ferroptosis in METTL3-suppressed cells. (C-F) Measurement of GSH/GSSH ratio (C), MDA levels (D), Fe^2+^ levels (E), and ROS levels (F) in control and METTL3 knockdown cells with or without IR treatment. (G-H) CCK-8 assay assessing the viability of HONE1 cells treated with the ferroptosis inducer Erastin (G) or the ferroptosis inhibitor ferrostatin-1 (H) at different doses. (I-J) Cell survival rates following irradiation at various doses, with corresponding D0, Dq, and N values depicted on the right. Erastin: 30 μM, ferrostatin-1: 1 μM. Data represent the mean ± SEM of three independent experiments, * *p* < 0.05; ** p < 0.01; *** p < 0.001.

This suggests a potential link between radiotherapy and ferroptosis, a regulatory form of cell death 24. Ferroptosis is an iron-dependent regulated cell death driven by lipid peroxidation and plays a significant role in radiation-induced cell death and tumor suppression 25. We investigated the role of METTL3 by conducting WB analysis on a stable METTL3 knockdown cell line. The results indicated that the expression levels of ferritin ferroptosis-related genes SLC7A11 and GPX4 were significantly reduced in METTL3 KD cells compared to the control group, with this difference becoming more pronounced post-combined irradiation (Figure 3A). SLC7A11 and GPX4 are key molecules closely linked to ferroptosis (iron-dependent cell death). Studies have shown that METTL3 regulates the mRNA methylation of SLC7A11, which indirectly influences the expression and function of GPX4 20, 26, 27. Transmission electron microscopy unveiled distinctive morphological features of ferroptosis in METTL3-KD cells, characterized by smaller mitochondria and increased membrane density (Figure 3B). A series of experiments were conducted in METTL3-KD cells to evaluate the levels of ferroptosis. Subsequent to METTL3 suppression, GSH/GSSH levels declined (Figure 3C), while MDA, Fe2+, and ROS levels escalated, with this effect becoming more pronounced post-irradiation (Figure 3D-F). Treatment of NPC cells with the ferroptosis inducer Erastin resulted in decreased cell viability, whereas cell viability increased following treatment with the ferroptosis inhibitor Fer-1 at different radiation doses (Figure 3G-H and Figure S1F-G). Colony formation assays demonstrated that Erastin treatment reversed METTL3 inhibition-induced cell death and post-irradiation growth suppression, whereas Fer-1 treatment yielded opposite outcomes in METTL3-overexpressing NPC cells (Figure 3I-J). These findings suggest that METTL3 promotes radioresistance by inhibiting NPC ferroptosis.

### 3.4 METTL3-mediated m6A modification of SLC7A11 promotes its stability in an IGF2BP2-dependent manner

**Figure 4:**
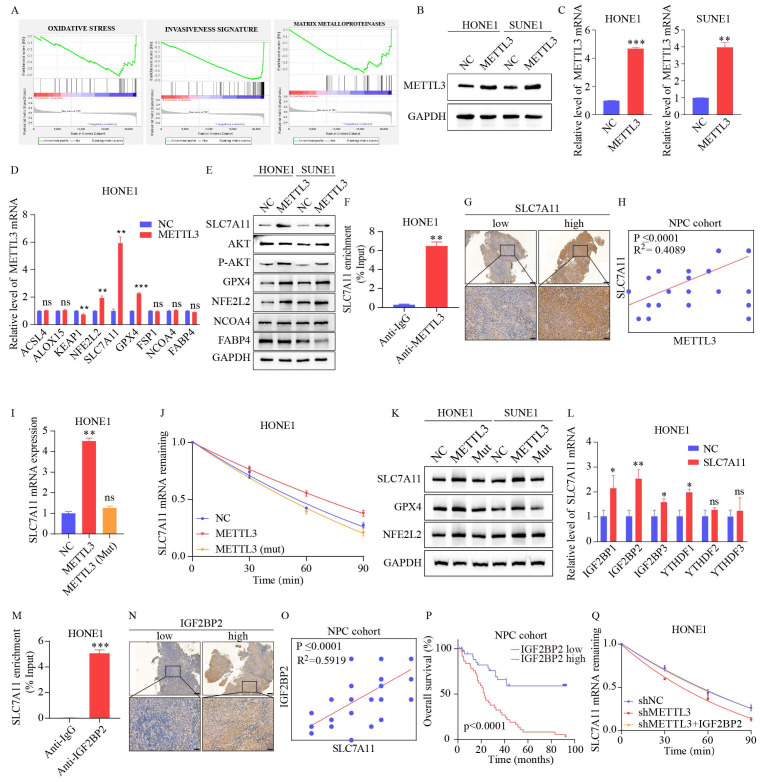
** METTL3 enhances the stability and expression of SLC7A11 mRNA through m6A modification recognized by IGF2BP2.** (A) GSEA of METTL3 overexpression phenotypes showing high/low expression signatures. (B-C) Validation of METTL3 overexpression by WB (B) and qPCR (C). (D-E) mRNA and protein expression of ferroptosis-related genes after METTL3 overexpression. (F) RIP-qPCR assay utilizes an METTL3-specific antibody and an IgG control antibody to detect the enrichment of METTL3 binding at the SLC7A11 m6A modification site. (G-H) Representative images (G) and scoring (H) of SLC7A11 expression in NPC tissues after IHC staining. (I) Confirmation of METTL3 overexpression in wild-type and mutant METTL3 cells. (J) qRT-PCR was used to assess the expression levels of SLC7A11 at specific time points after Act D (2 µg/mL) treatment in HONE1 wild-type METTL3, mutant METTL3, and their respective control cells. (K) Expression changes of ferroptosis-related genes in wild-type and mutant METTL3 cells confirmed by WB. (L) Expression of six m6A reader enzymes detected by qPCR in control and METTL3-overexpressing HONE1 cells. (M) RIP-qPCR assay utilizes an IGF2BP2-specific antibody and an IgG control antibody to detect the enrichment of IGF2BP2 binding at the SLC7A11 m6A modification site. (N-O) Representative images (N) and scoring (O) of SLC7A11 expression in NPC tissues after IHC staining. (P) Kaplan-Meier analysis of overall survival rates in NPC patients with high/low expression of IGF2BP2. (Q) qRT-PCR was used to assess the expression levels of SLC7A11 at specific time points after treatment with Act D (2 µg/mL) in HONE1 control cells, METTL3 knockdown cells, and METTL3 knockdown cells transfected with IGF2BP2. Data represent the mean ± SEM of three independent experiments, * *p* < 0.05; ** p < 0.01; *** p < 0.001.

To clarify the potential mechanism of METTL3 in ferroptosis, Gene Set Enrichment Analysis (GSEA) was performed based on the GSE12452 dataset, which indicated that METTL3 expression was significantly associated with ferroptosis-related pathways, including the oxidative stress pathway (Figure 4A). We first established stable METTL3-overexpressing NPC cells (Figure 4B-C). Based on previous studies 28-33, nine oxidative stress-related genes were selected for validation. The results showed that the expression levels of NFE2L2, SLC7A11, GPX4, and phosphorylated AKT were upregulated in METTL3-overexpressing cells, with the most significant expression of SLC7A11 (Figure 4D-E and S1H). As shown in Figures 4F and S1I, the METTL3-specific antibody significantly enriched SLC7A11 mRNA in the RIP-qPCR experiment, compared to the IgG control antibody. Furthermore, IHC revealed that SLC7A11 expression was positively correlated with METTL3 expression (Figure 4G-H).

Given that m6A modification positively regulated the mRNA level of SLC7A11, we investigated whether m6A modification affected the stability of SLC7A11 mRNA. The NPC cells were treated with actinomycin D, a transcription inhibitor, for the indicated times. The level of SLC7A11 mRNA was shown to be highly stable with METTL3 overexpression, while the effect was reversed by METTL3 mutation (Figure 4I-K and S1J). A recent study reported that IGF2BPs and YTHDFs are distinct families of m6A readers that target thousands of mRNA transcripts through the recognition of the m6A motif 9, 34. The effects of IGF2BPs and YTHDFs on SLC7A11 mRNA stabilization were further explored. We transiently transfected IGF2BPs and YTHDFs plasmids to detect SLC7A11 expression. Only overexpression of IGF2BP2 markedly enhanced SLC7A11 mRNA expression (Figure 4L and S1K). The IGF2BP2-specific antibody notably enriched SLC7A11 mRNA in the RIP-qPCR assay, in contrast to the IgG control antibody (Figure 4M and S1L). Additionally, a significant correlation was observed between the expression of IGF2BP2 and SLC7A11 (r²=0.5919; p<0.0001, Figure 4N-O and S1M). Based on the median of IGF2BP2 immunohistochemistry scores, patients with expression levels higher than the median were classified as the "high expression" group, while those with levels below the median were classified as the "low expression" group. Kaplan-Meier analysis indicates that high IGF2BP2 expression correlates with poor prognosis in patients with NPC (Figure 4P and S1N). The reduction of SLC7A11 mRNA caused by METTL3 knockdown could be rescued by IGF2BP2 (Figure 4Q and S1O). Together, our findings suggest that METTL3-mediated m6A modification maintains the stability of SLC7A11 expression in an IGF2BP2-dependent manner.

### 3.5 The elevation of METTL3 accelerates NPC progression by upregulating SLC7A11 expression

**Figure 5:**
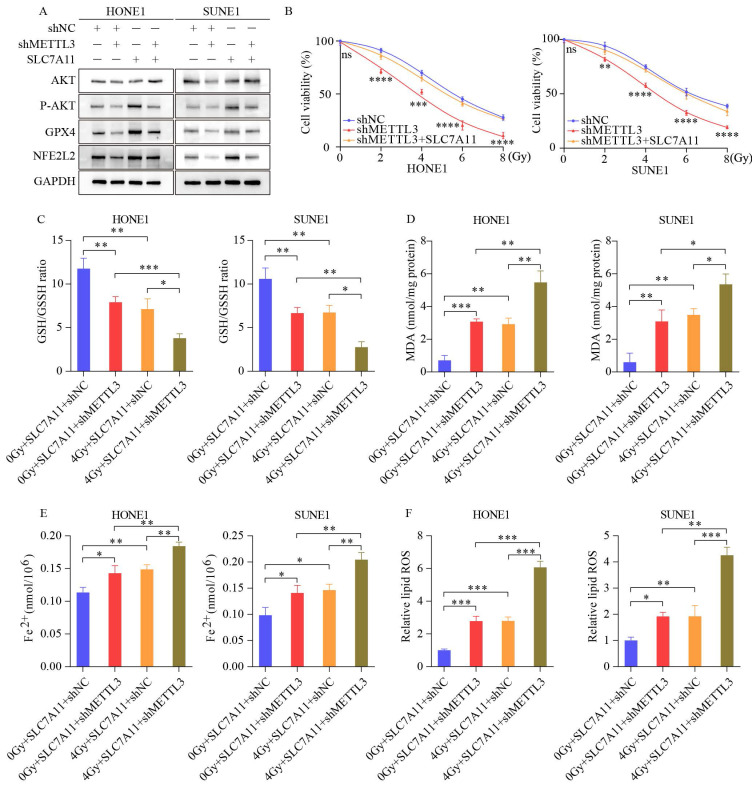
** SLC7A11 overexpression reverses ferroptosis-related effects induced by METTL3 knockdown in NPC cells.** (A) SLC7A11 overexpression in METTL3 knockdown cells restores the protein expression of phosphorylated AKT, GPX4, and NFE2L2. (B) Enhanced growth capacity of nasopharyngeal carcinoma cells upon SLC7A11 expression in METTL3 knockdown cells. (C-F) Measurement of GSH/GSSH ratio (C), MDA (D), Fe²⁺ levels (E), and ROS levels (F) in METTL3 knockdown cells overexpressing SLC7A11 with or without IR. Data represent the mean ± SEM of three independent experiments, * *p* < 0.05; ** p < 0.01; *** p < 0.001.

Western blot assays revealed that SLC7A11 overexpression counteracted the reduction in GPX4, NFE2L2, and phosphorylated AKT levels induced by METTL3 knockdown (Figure 5A). CCK8 assays demonstrated that SLC7A11 overexpression rescued the cell viability of METTL3-KD cells (Figure 5B). Furthermore, SLC7A11 overexpression reversed the decreased GSH/GSSH ratio (Figure 5C) and the increased levels of MDA, Fe2+, and ROS induced by METTL3 inhibition (Figure 5D-F). Our findings indicate that METTL3 inhibits radiotherapy sensitivity by upregulating SLC7A11 expression and mitigating ferroptosis, thereby accelerating the malignant progression of NPC.

### 3.6 METTL3 collaborates with ferroptosis inducers to regulate radiosensitivity *in vivo*

**Figure 6:**
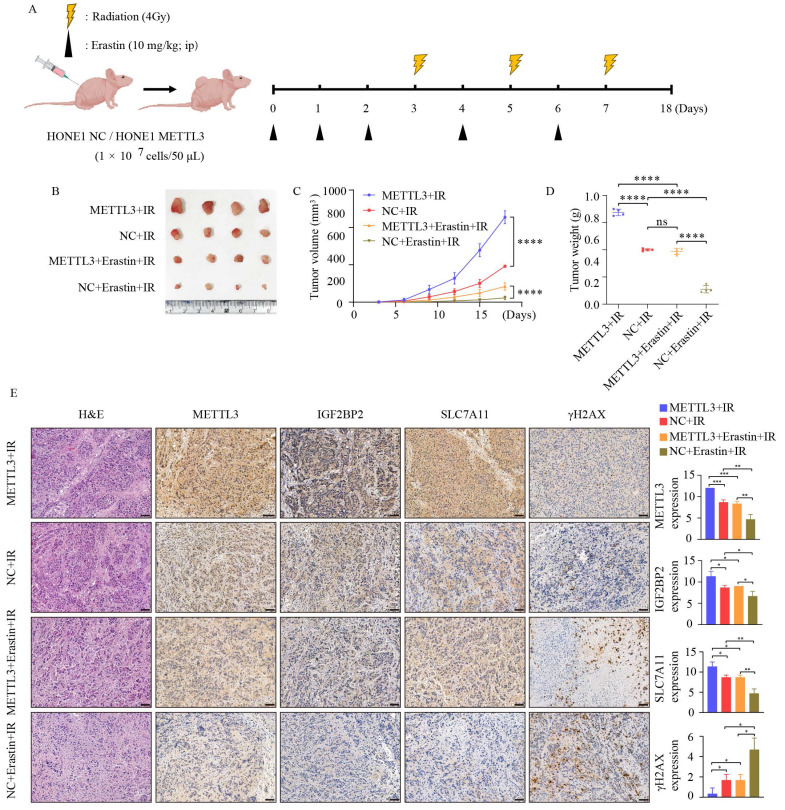
** METTL3 promotes radioresistance in NPC *in vivo*.** (A) Schematic representation of the experimental design and workflow for subcutaneous tumor transplantation in nude mice. (B) Tumor growth status in each group after 18 days of treatment (n=4). (C) Tumor volume. (D) Tumor weight. (E) Representative images of H&E staining and IHC staining for METTL3, IGF2BP2, SLC7A11, and γ-H2AX protein expression in subcutaneously transplanted tumors. Scale bar: 50 µm. Quantitative data from three repeated immunohistochemical images are presented on the right. Data represent the mean ± SEM of three independent experiments, * *p* < 0.05; ** p < 0.01; *** p < 0.001.

To further investigate whether METTL3 enhances the radioresistance of NPC *in vivo*, we established subcutaneous xenografts in nude mice, as depicted in Figure 6A. Compared to the control group, METTL3 overexpression significantly increased the volume and weight of the tumors, regardless of the presence of IR and Erastin. Moreover, the combination treatment with IR and Erastin resulted in lower tumor volume and weight compared to the IR group. Importantly, the combination therapy of IR and Erastin demonstrated superior efficacy, overcoming METTL3-induced radiotherapy resistance (Figure 6B-D). Figure 6E illustrates that radiation combined with the ferroptosis inducer Erastin led to decreased expression of METTL3, IGF2BP2, and SLC7A11, while upregulating γ-H2AX expression. In summary, METTL3 in combination with ferroptosis inducers can promote radiotherapy sensitivity *in vivo*. These data suggest that the METTL3/IGF2BP2/SLC7A11 axis promotes NPC radioresistance by inhibiting NPC ferroptosis.

## 4. Discussion

Due to its special anatomical location and nonspecific symptoms, most NPC cases are diagnosed at advanced stages. For advanced-stage patients, radiotherapy combined with chemotherapy has become the primary treatment strategy 35. Despite advancements in treatment and technology, some patients still experience residual tumors after radiotherapy or develop local recurrence, with complications and mortality being prominent. The inherent radiotherapy resistance of NPC cells may be a crucial factor contributing to treatment failure 1, 36. Hence, there is an urgent need to elucidate the molecular mechanisms underlying radioresistance and to identify potential therapeutic targets. In this study, we identified that the m6A level is significantly increased due to the upregulation of methyltransferase METTL3 in NPC. This upregulation stimulates the m6A modification of SLC7A11 mRNA. The m6A reader IGF2BP2 then directly binds to the m6A site on SLC7A11 mRNA and maintains its stability, which inhibits ferroptosis in NPC. This process promotes the progression of NPC and leads to a worse clinical prognosis.

Among m6A modification regulatory factors, METTL3 has been thoroughly and extensively studied in the progression of various cancer types, with multiple studies confirming METTL3 as an independent prognostic factor significantly associated with survival rates in NPC patients 37, 38. Previous reports have highlighted the role of METTL3 in DNA damage 39, 40. It is generally believed that ionizing radiation can induce DNA double-strand breaks (DSBs), leading to cell death in tumor cells. However, DNA DSBs also activate complex and highly regulated DNA damage response (DDR) signaling, resulting in radiotherapy resistance in cancer cells 41. Currently, the relationship and underlying mechanisms between METTL3 and radioresistance in NPC remain largely unexplored. In this study, we initially analyzed the high expression of METTL3 in NPC tissues. TCGA database revealed that METTL3 was significantly upregulated in various cancer types. Furthermore, we found that METTL3 is associated with poor prognosis in NPC patients, correlating with clinical characteristics. *In vitro* and *in vivo* assays found that ectopic expression of METTL3 regulated radioresistance through ferroptosis-related pathways.

Ferroptosis is a crucial pathway through which radiation induces tumor cell death. Radiation-induced reactive oxygen species (ROS) can lead to lipid peroxidation of cell membranes, ultimately triggering ferroptosis 42. Irradiation also induces adaptive responses involving SLC7A11 or GPX4, inhibiting radiation-induced ferroptosis and promoting tumor cell survival, leading to radioresistance 25. Previous studies have reported that METTL3 is associated with lipid peroxidation and ferroptosis 27. However, there is no literature reporting the relationship among METTL3, radioresistance, and ferroptosis in NPC. Functional experiments indicated that inhibiting METTL3 can lead to increased lipid peroxidation and eventual ferroptosis in NPC cells, with or without irradiation. Overexpression of METTL3 followed by treatment with the ferroptosis inducer Erastin promoted the radiosensitivity of NPC cells. Thus, METTL3-induced ferroptosis in NPC cells may play a crucial role in radiotherapy resistance. We investigated the potential mechanisms by which METTL3 regulates radiotherapy resistance in NPC. By integrating RNA-seq and m6A-seq data, we identified SLC7A11 as a potential downstream target of METTL3 in NPC. SLC7A11 has been validated as a potential biomarker closely associated with tumorigenesis 43, 44. SLC7A11 promotes glutathione (GSH) synthesis and reduces the generation of lipid hydroperoxides (L-OOH) to protect tumor cells from oxidative stress and ferroptosis 45. Our data revealed a significant positive correlation between the expression levels of SLC7A11 and METTL3, with high levels of SLC7A11 indicating poor prognosis.

Moreover, we observed that overexpression of METTL3 leads to upregulation of the phosphorylation levels of NFE2L2, GPX4, and AKT. Nuclear factor erythroid 2-related factor 2 (NFE2L2) is considered the major transcription factor regulating stress-induced transcription of SLC7A11. NFE2L2 primarily mediates transcriptional programs in response to oxidative stress, regulating SLC7A11 expression and thereby altering cellular sensitivity to ferroptosis 46. Glutathione peroxidase 4 (GPX4) serves as a major oxidative-reduction enzyme utilizing GSH as a reducing agent to detoxify lipid peroxides 47. Multiple studies have demonstrated that AKT signaling promotes radioresistance in tumor cells by modulating DNA damage repair processes 48-50. In this study, we confirmed a novel regulatory pathway whereby METTL3 mediates m6A modification-dependent stability of SLC7A11 mRNA via IGF2BP2, sustaining SLC7A11 expression in NPC cells. The radioresistance and ferroptosis induced by METTL3 could be partially reversed by downregulation of SLC7A11. The combined application of the ferroptosis inducer Erastin and radiation may serve as a potential therapeutic avenue for NPC patients exhibiting METTL3 overexpression.

In summary, this study elucidates the oncogenic role of METTL3 in the radioresistance and progression of NPC. Mechanistically, the METTL3/IGF2BP2/SLC7A11 axis fosters radioresistance and inhibits ferroptosis, thereby facilitating the progression of NPC.

The relatively small sample size of the animal experiments in this study may limit the statistical power of the results. Some heterogeneity exists between groups, potentially due to individual biological differences and minor variations in experimental conditions. However, we used appropriate statistical methods to control these factors, and the results align with existing literature 26, 27, 51-54. Increasing the sample size in future studies will be essential to improve the robustness and generalizability of the findings. Furthermore, future research should emphasize combining the development and clinical application of METTL3 inhibitors to facilitate their translation into radiosensitizers. In personalized treatment, integrating METTL3 and SLC7A11 expression levels for patient stratification may offer more precise treatment plans for nasopharyngeal carcinoma patients. Additionally, combining radiotherapy with ferroptosis inducers like Erastin could become a novel therapeutic approach, especially for patients with resistant or recurrent disease. Validating the therapeutic potential and prognostic value of the METTL3/IGF2BP2/SLC7A11 axis via clinical trials will be crucial for translating this research into clinical practice.

## Supplementary Material

Supplementary figure.

Supplementary tables.

## Abbreviations

NPC: nasopharyngeal carcinoma
m6A: N6-methyladenosine
METTL3: Methyltransferase 3
SLC7A11/ xCT: Solute carrier family 7 member 11
GPX4: Glutathione peroxidase 4
NFE2L2: NFE2 like bZIP transcription factor 2
IGF2BP2: Insulin like growth factor 2 mRNA binding protein 2
GSH: Glutathione
GSSH: Oxidized glutathione
MDA: Malondialdehyde
ROS: Reactive Oxygen Species
GSEA: Gene Set Enrichment Analysis
EBV: Epstein-Barr virus
TCGA: The Cancer Genome Atlas
qRT-PCR: Quantitative Real-time PCR
IMRT: Intensity Modulated Radiotherapy
DDR: DNA damage response
DSB: Double Strand Break
PUFA: Polyunsaturated fatty
DNA-PKcs: DNA-dependent protein kinase catalytic subunit
