# Supplementary material for: METTL3-mediated m6A modification of SLC7A11 enhances nasopharyngeal carcinoma radioresistance by inhibiting ferroptosis
Source: Int J Biol Sci. 2025 Feb 10;21(4):1837–51. doi: 10.7150/ijbs.100518 (PMC11844296; doi:10.7150/ijbs.100518)
Supplement: Supplementary file 1 — Supplementary figure. [file ijbsv21p1837s1.pdf]

Figure Legend

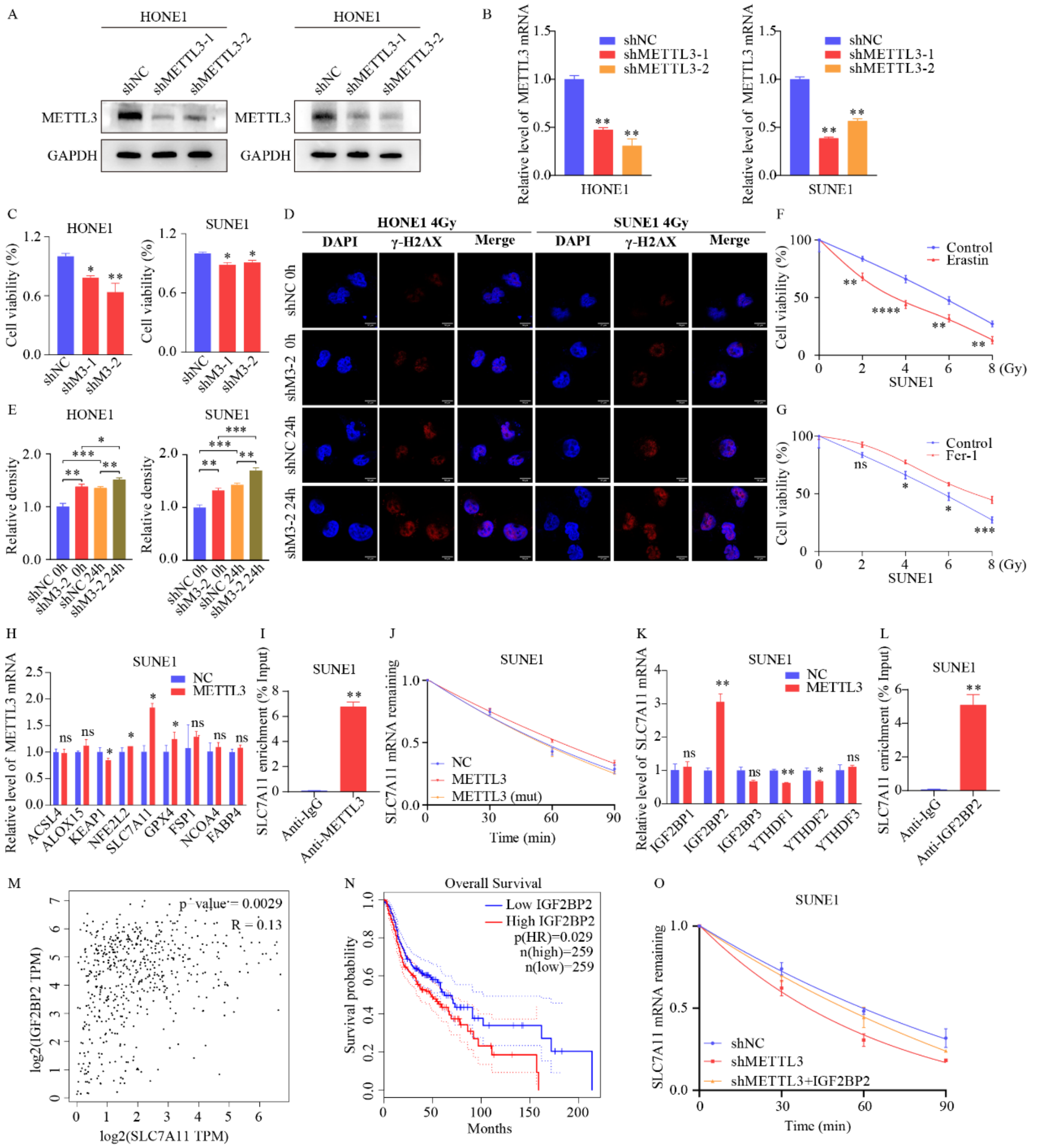

Figure S1. (A-B) WB (A) and qRT-PCR (B) assays of METTL3 expression in cells with stable METTL3 knockdown. (C) Cell viability was evaluated in HONE1 and SUNE1 cells that were transfected with shNC, shM3-1, and shM3-2. (D-E) IF staining (D) and relative density analysis (E) of γ-H2AX in in HONE1 and SUNE1 cells after 4 Gy irradiation at 0 and 24 hours. (F-G) Cell viability of HONE1 cells treated with the ferroptosis inducer Erastin (F) or the ferroptosis inhibitor ferrostatin-1 (G) at different

doses, as measured by CCK-8 assay. (H) mRNA expression of ferroptosis-related genes in SUNE1 cells overexpressing METTL3. (I) RIP-qPCR assay utilizes an METTL3-specific antibody and an IgG control antibody to detect the enrichment of METTL3 binding at the SLC7A11 m6A modification site. (J) qRT-PCR was used to assess the expression levels of SLC7A11 at specific time points after Act D (2 μg/mL) treatment in SUNE1 wild-type METTL3, mutant METTL3, and their respective control cells. (K) Expression of six m6A reader enzymes in control cells and SUNE1 cells overexpressing METTL3 detected by qPCR. (L) RIP-qPCR assay utilizes an IGF2BP2-specific antibody and an IgG control antibody to detect the enrichment of IGF2BP2 binding at the SLC7A11 m6A modification site. (M) Correlation between IGF2BP2 and SLC7A11 expression in NPC specimens from the TCGA database, analyzed using Spearman's test. (N) Overall survival rates of NPC patients with high or low expression of IGF2BP2 in the TCGA-HNSC database. (O) qRT-PCR was used to assess the expression levels of SLC7A11 at specific time points after treatment with Act D (2 μg/mL) in SUNE1 control cells, METTL3 knockdown cells, and METTL3 knockdown cells transfected with IGF2BP2.
